# E2F4 Mediates Mitophagy to Inhibit Ferroptosis in Esophageal Cancer Cells by Activating GPR176

**DOI:** 10.1155/humu/9418012

**Published:** 2026-05-30

**Authors:** Weilang Xu, Fang Su, Luxuan Xie, Tao Ding, Xiao Qi, Peiqiang Chen, Zaiyuan Ye, Wenfa Lin

**Affiliations:** ^1^ Department of Gastrointestinal Surgery, The Second Affiliated Hospital of Zhejiang Chinese Medical University, Hangzhou, Zhejiang, China, z2hospital.com; ^2^ Department of Surgery, Shulan (Hangzhou) Hospital, Affiliated to Shulan International Medical College, Zhejiang Shuren University, Hangzhou, Zhejiang, China, zjsru.edu.cn; ^3^ Graduate School, Zhejiang Chinese Medical University, Hangzhou, Zhejiang, China, zcmu.edu.cn; ^4^ Key Laboratory of Gastroenterology of Zhejiang Province, Zhejiang Provincial People’s Hospital, Affiliated People’s Hospital, Hangzhou Medical College, Hangzhou, Zhejiang, China, hznu.edu.cn; ^5^ Department of Gastroenterology, The Third Affiliated Hospital of Zhejiang Chinese Medical University, Hangzhou, Zhejiang, China, zjztyy.com; ^6^ General Surgery, Cancer Center, Department of Gastrointestinal and Pancreatic Surgery, Zhejiang Provincial People’s Hospital, The Affiliated People’s Hospital of Hangzhou Medical College, Hangzhou, Zhejiang, China, hospitalstar.com

**Keywords:** E2F4, esophageal cancer, ferroptosis, GPR176, mitophagy

## Abstract

Esophageal cancer (EC) is driven by complex dysregulated molecular networks, and ferroptosis—an iron‐dependent, non‐apoptotic form of regulated cell death—has emerged as a critical modulator of tumorigenesis. However, the functional contribution and mechanistic basis of GPR176 in ferroptosis regulation during EC progression remain largely unexplored. Here, we integrated computational and experimental approaches to delineate the role of GPR176 and its upstream regulator E2F4 in EC ferroptosis. Bioinformatic analysis revealed consistent upregulation of both GPR176 and E2F4 in EC tissues, which was further confirmed by molecular validation. Functional assays demonstrated that GPR176 overexpression conferred resistance to ferroptosis in EC cells, as reflected by reduced malondialdehyde, intracellular Fe^2+^, and lipid reactive oxygen species (ROS) accumulation, alongside altered expression of core ferroptosis mediators. This protective effect was associated with the suppression of mitophagy, as indicated by alterations in mitochondrial function and autophagy‐related markers. Mechanistically, we demonstrated that E2F4 directly binds to the GPR176 promoter and transcriptionally activates its expression. Rescue experiments further validated that GPR176 overexpression abrogated the enhanced mitophagy and ferroptosis induced by E2F4 depletion. Collectively, our findings define an E2F4/GPR176/mitophagy axis that acts to suppress ferroptosis in EC, highlighting this pathway as a novel therapeutic target for inducing ferroptosis in EC intervention.

## 1. Introduction

Esophageal cancer (EC) represents a globally prevalent malignancy of the digestive system, holding the seventh position in incidence and the sixth in mortality worldwide [1]. Despite continuous advances in diagnostic and therapeutic techniques, the five‐year survival rate for patients generally remains under 20%, showing little substantial change in recent decades [2]. The disease primarily manifests as either squamous cell carcinoma or adenocarcinoma, with established risk factors encompassing tobacco use, alcohol intake, gastroesophageal reflux, and certain dietary patterns [3]. Standard treatment modalities for EC include surgical resection, radiotherapy, chemotherapy, supplemented by newer approaches such as targeted and immunotherapies [4, 5]. Even with refinements in multimodal treatment strategies, overall outcomes are often unfavorable, largely due to local recurrence and distant metastasis [6]. Given their indispensable role in advancing patient prognoses, the comprehensive delineation of the molecular drivers governing EC pathogenesis and the discovery of innovative therapeutic targets hold profound academic and clinical significance.

In recent cancer research, ferroptosis, a form of iron‐dependent programmed cell death, has gained significant attention as a promising therapeutic avenue [7]. Its defining hallmarks include the abnormal accumulation of intracellular lipid peroxides and the functional suppression of GPX4 [8]. Notably, triggering ferroptosis has been shown to impede the growth of multiple cancer types, highlighting the modulation of ferroptotic pathways as a potential strategy for cancer treatment [9]. For example, in EC, stem cells can evade this form of death by activating the Hsp27‐GPX4 signaling axis, indicating that therapeutic intervention targeting this pathway may offer a novel approach to treatment [10]. Of related significance, autophagy, specifically mitophagy, is essential for cellular homeostasis and death regulation [11]. Emerging evidence indicates a dynamic and bidirectional interplay between mitophagy and ferroptosis. While mitophagy can suppress ferroptosis by eliminating damaged mitochondria, it may, in certain contexts, also facilitate ferroptotic cell death [12, 13]. Despite these general insights, the precise regulatory networks and mechanistic interactions linking ferroptosis and mitophagy in EC are not well‐defined, necessitating comprehensive investigation.

GPR176, a protein belonging to the GPCR superfamily, participates in modulating numerous intracellular signaling cascades [14]. Research has indicated the aberrant expression of GPR176 in several cancers and its contribution to tumor pathogenesis [15, 16]. For example, in gastric cancer, GPR176 upregulation correlates with unfavorable clinical outcomes and alterations in the tumor immune microenvironment, highlighting its potential as both a prognostic indicator and a therapeutic target [17]. These findings suggest that GPR176 may play important roles in the progression of digestive system malignancies. Notably, certain GPCR family members can affect susceptibility to ferroptosis by regulating cellular metabolism and oxidative stress [18]. Given that GPR176 is a member of the GPCR superfamily and has been implicated in digestive system malignancies, it is reasonable to speculate that GPR176 may also participate in the regulation of ferroptosis in EC. However, the expression profile, biological functions, and potential involvement of GPR176 in EC, particularly regarding its influence on ferroptosis and disease progression, remain unknown.

This study reveals the high expression of GPR176 in EC and its suppression of ferroptosis in EC cells. E2F4 transcriptionally activates GPR176 and induces ferroptosis resistance in EC cells by inhibiting the mitophagy pathway. This study elucidates a novel regulatory pathway for ferroptosis in EC and proposes a potential strategy to reverse EC treatment resistance by targeting the E2F4‐GPR176‐mitophagy axis.

## 2. Materials and Methods

### 2.1. Patients and Samples

From November 2022 to September 2024, 10 paired EC tissues and adjacent noncancerous tissues (at least 5 cm away from the tumor tissues) were collected by surgical resection at Shulan (Hangzhou) Hospital (No. KY2026033). None of the patients received radiotherapy or chemotherapy before surgery. All samples were immediately placed in liquid nitrogen and stored after resection. This study was approved by the Ethics Committee of Shulan (Hangzhou) Hospital, with written informed consent obtained from all patients.

### 2.2. EC Data Retrieved From Public Databases

Transcriptome data for EC were obtained from TCGA (13 normal, 185 tumor) and were subjected to differential expression analysis with the “Limma” R package (|*l* *o* *g* *F* *C*| > 0.585, FDR < 0.05). The correlation between GPR176 and key ferroptosis‐related genes was assessed via bioinformatic approaches. Potential upstream transcription factors (TFs) of GPR176 were screened via the hTFtarget database (https://guolab.wchscu.cn/hTFtarget/) and underwent Pearson correlation analysis to evaluate their relationships with GPR176 expression; their binding sites were predicted on the JASPAR website (https://jaspar.elixir.no/).

### 2.3. Cell Culture

The EC cell lines KYSE450 (CTCC‐001‐0746, Meisen Cell, China), TE‐1 (CTCC‐007‐0592, Meisen Cell, China), and ECA109 (AW‐CH0082, Anweisci, China) were cultured in RPMI‐1640 medium (Gibco, United States). Normal esophageal epithelial cells Het‐1A (CTCC‐001‐0223, Meisen Cell, China) were cultured in BEGM (Meisen Cell, China). Two hundred and ninety‐three T cells (CRL‐3216, ATCC, United States) were cultured in DMEM (Gibco, United States). All media contained 10% FBS (Thermo Fisher, United States) and 1% penicillin–streptomycin (Thermo Fisher, United States). Culture continued at 37°C with 5% CO₂ in a humidified incubator. Fetal bovine serum (FBS) was heat‐inactivated at 56°C for 30 min before use.

### 2.4. Plasmid Transfection and Reagent Treatment

According to the basal expression level of GPR176 in EC cells, ECA109 cells were used for knockdown experiments, whereas KYSE450 cells were used for overexpression experiments. For GPR176 functional assays, ECA109 cells were transfected with sh‐NC or sh‐GPR176, and KYSE450 cells were transfected with oe‐NC or oe‐GPR176. For the mitophagy rescue experiment, KYSE450 cells were divided into three groups: oe‐NC + DMSO, oe‐GPR176 + DMSO, and oe‐GPR176 + olaparib. For the upstream rescue experiment, ECA109 cells were divided into three groups: sh‐NC + oe‐NC, sh‐E2F4 + oe‐NC, and sh‐E2F4 + oe‐GPR176. In experiments involving autophagy observation, cells were co‐transfected with the GFP‐LC3 plasmid as indicated. sh‐NC, sh‐GPR176, oe‐NC, oe‐GPR176, sh‐E2F4 (GeneSmart, China), and GFP‐LC3 plasmid (Beyotime, China) were transfected into EC cells with HighGene transfection reagent (ABclonal, China), with transfection efficiency validated by qRT‐PCR. The mitophagy inducer olaparib (MedChemExpress, United States) was dissolved in DMSO (Beyotime, China) and applied at 2 *μ*M for 24 h.

### 2.5. qRT‐PCR

Total RNA was extracted from harvested cells with TRIzol reagent (15596026CN, Invitrogen, United States) and quantified for purity and concentration on a spectrophotometer (Thermo Fisher, United States). Following cDNA synthesis via reverse transcription with the PrimeScript RT reagent kit (Takara, Japan, RR037A), qRT‐PCR was run on an ABI 7500 system (Invitrogen, United States) with TB Green Premix Ex Taq II (RR820A, Takara, Japan). The relative expression of target genes was normalized to *β*‐actin and calculated by the 2^−*ΔΔ*CT^ method, with specific primer sequences provided in Table S1.

### 2.6. Western Blot (WB)

Following a 48‐h transfection, EC cells from each group were collected and lysed on ice for 10 min in RIPA buffer (Beyotime, China) containing both 1× protease and 1× phosphatase inhibitors (Beyotime, China). After centrifugation (12,000 ×*g*, 15 min, 4°C), the supernatant was collected and quantified for protein concentration with a BCA assay kit (P0398S, Beyotime, China). Equal protein amounts were separated by 10% SDS‐PAGE (Solabao, China), transferred to PVDF membranes (Sangon Biotech, China), and blocked with 5% nonfat milk (Beyotime, China). The membranes were first incubated with primary antibodies overnight at 4°C. After washing three times with TBST (Beyotime, China; each 5 min), they were incubated with HRP‐conjugated secondary antibodies for 2 h at room temperature. Protein bands were detected with ECL reagent (Bio‐Rad, United States) and captured on a gel imaging system (Bio‐Rad, United States). Specific information for all antibodies is detailed in Table S2.

### 2.7. CCK‐8 Viability Assay

To assess cell proliferation, ECA109 and KYSE450 cells were transfected with the respective plasmids in 12‐well plates upon reaching approximately 80% confluency. Following transfection, cells were transferred to 96‐well plates at 2 × 10^3^ cells/well. At 0, 24, 48, and 72 h, 10 *μ*L of CCK‐8 reagent (EZB, United States) was introduced to each well and maintained at 37°C for 2 h. Optical density (OD) at 450 nm was read with a microplate reader (Bio‐Rad, United States).

### 2.8. Measurement of MDA and Fe^2+^

To evaluate intracellular lipid peroxidation, MDA levels were quantified with a commercially available assay kit (S0131S, Beyotime, China). Following a wash with ice‐cold PBS (Beyotime, China), EC cells were lysed in lysis buffer (Beyotime, China) on ice. The resulting lysates underwent centrifugation at 12,000 ×*g* for 10 min at 4°C. The clarified supernatant was reacted with a TBA solution, which involved heating at 95°C for 30 min and centrifugation at 1000 ×*g* after cooling. The OD of the final supernatant was recorded at 532 nm on a Bio‐Rad microplate reader (United States).

To measure Fe^2+^ levels, differently treated EC cells were harvested and rinsed with cold PBS (Beyotime, China). Intracellular Fe^2+^ concentration was determined with an iron assay kit (ab83366, Abcam, United Kingdom). In this procedure, cell samples were mixed with the provided buffer for 30 min, followed by a 60‐min reaction with an iron‐specific probe. OD readings were taken at 593 nm with the Bio‐Rad microplate reader (United States).

### 2.9. Lipid ROS Assessment

Lipid ROS levels in EC cells were analyzed with BODIPY 581/591 C11 (D3861, Thermo Fisher, United States). BODIPY 581/591 C11 (2 *μ*M) was added 30 min prior to cell collection to measure lipid ROS. Analysis was performed on a NovoCyte flow cytometer (Agilent, United States).

### 2.10. Confocal Detection of LC3 Puncta Formation

Transfected cells (1 × 10^6^ cells/well) in 6‐well plates underwent transfection with the GFP‐LC3 plasmid for 48 h and fixation in 4% paraformaldehyde (Beyotime, China), followed by three washes with PBS (Beyotime, China). Cell nuclei were stained with DAPI (Beyotime, China) for 2–3 min before imaging under a TCS SP2 confocal fluorescence microscope (Leica, Germany).

### 2.11. JC‐1 Assay for Mitochondrial Membrane Potential (MMP)

JC‐1 staining working solution was prepared with the MMP assay kit (C2006, Beyotime, China). After medium aspiration, each well received 1 mL of JC‐1 staining solution for 20‐min incubation at 37°C. Cells were collected by trypsinizing, washed twice with 1× JC‐1 staining buffer, and resuspended in 500 *μ*L of PBS (Beyotime, China) prior to analysis with a NovoCyte flow cytometer (Agilent, United States).

### 2.12. Immunohistochemistry (IHC)

Tumor tissues from surgical resection were fixed in 4% paraformaldehyde (Beyotime, China) for 24 h, embedded in paraffin, and sliced into 4 *μ*m‐thick sections. These paraffin sections were deparaffinized in xylene (Sigma‐Aldrich, United States) and rehydrated through a graded ethanol series (100%, 95%, 85%, 75%; Sigma‐Aldrich, United States). For antigen retrieval, sections were heated in an EDTA‐based solution (Beyotime, China) in a microwave, cooled to room temperature, and washed three times with PBS (Beyotime, China). Endogenous peroxidase was quenched with 3% H₂O₂ (Beyotime, China) for 10 min, followed by three PBS washes. Nonspecific binding was blocked with 5% BSA (Beyotime, China) for 30 min at room temperature. After removing the blocking solution, sections were treated with primary antibodies and left to incubate overnight at 4°C. The following day, after eliminating unbound primary antibody through PBS washes, sections were exposed to HRP‐conjugated secondary antibodies for 1 h at room temperature and washed with PBS. Chromogenic visualization was achieved with DAB substrate (DAKO, Denmark). After counterstaining nuclei with hematoxylin (Beyotime, China), sections were imaged under an optical microscope (Leica, Germany). Information regarding the specific primary antibodies used is detailed in Table S3.

### 2.13. Dual‐Luciferase Reporter Assay

The synthesized pGL3.0‐GPR176‐promoter‐WT and pGL3.0‐GPR176‐promoter‐MUT vectors, or sh‐NC/sh‐E2F4, were co‐transfected into 293 T cells with HighGene transfection reagent. Cells were lysed with lysis buffer 48 h after transfection. Luciferase activity was measured with the Dual‐Luciferase Reporter Assay Kit (E1910, Promega, United States).

### 2.14. Chromatin Immunoprecipitation (ChIP)

For the ChIP assays, the protocol employed was from the EZ‐ChIP kit (Merck Millipore, Germany, 17‐371). EC lysates were collected and underwent immunoprecipitation overnight at 4°C with a ChIP‐validated rabbit anti‐E2F4 antibody (40291, Cell Signaling Technology, United States) or anti‐IgG control (3900S, CST, United States). Protein A/G magnetic beads (Beyotime, China) were introduced and the mixture was incubated for 4 h. DNA was extracted and purified from the protein–DNA complexes with a DNA purification kit (K0512, Thermo Fisher Scientific, United States). The purified DNA was quantified via qRT‐PCR. The sequences of the primers applied in the ChIP‐qPCR are detailed in Table S4. The amplified fragment length ranged from 120 to 150 bp, and the annealing temperature was set at 60°C.

### 2.15. Statistical Analysis

All experiments were performed with at least three biological replicates and independently repeated at least three times. Data are presented as mean ± standard deviation (SD) and were analyzed in GraphPad Prism 9.0 (GraphPad Software, United States). Comparisons between two groups were conducted using Student’s *t* test, while comparisons among multiple groups were performed using one‐way ANOVA followed by Tukey’s post hoc test. *p* < 0.05 was considered statistically significant.

## 3. Results

### 3.1. GPR176 is Highly Expressed in EC and Involved in the Ferroptosis Pathway of EC Tumors

**Figure 1:**
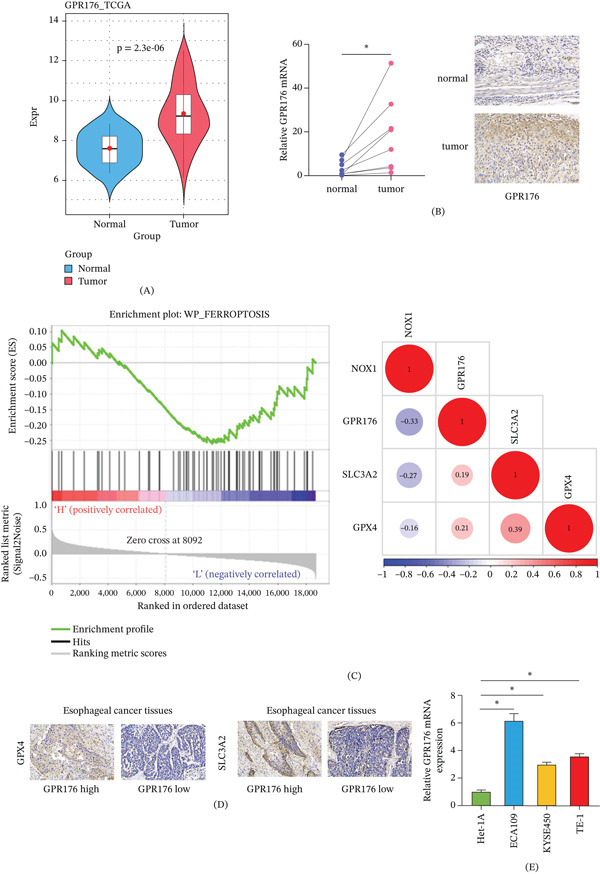
GPR176 is highly expressed in EC and participates in the ferroptosis pathway of EC tumors. (A) Expression of GPR176 in EC tissues analyzed via bioinformatics; (B) qRT‐PCR and IHC detection of GPR176 expression in patient tissues; (C) Bioinformatic analysis of GPR176‐enriched pathways and its correlation with key ferroptosis genes; (D) IHC analysis of GPX4 and SLC3A2 expression in tumor tissues from patients with EC with high or low GPR176 expression; (E) qRT‐PCR analysis of GPR176 expression in normal human esophageal cells (Het‐1A) and EC cell lines (ECA109, KYSE450, TE‐1); Data are presented as mean ± SD from three independent experiments. ns, not significant; ∗*p* < 0.05.

To investigate the expression of GPR176 in EC, bioinformatic analysis was performed. As shown in Figure 1A, GPR176 was significantly upregulated in tumor tissues from patients with EC. To validate this, tumor tissues and paired adjacent normal tissues from 10 patients with EC were collected. Figure 1B shows that GPR176 expression was markedly increased at both the mRNA and protein levels in tumor tissues, as determined by qRT‐PCR and IHC. As presented in Figure 1C, pathway enrichment analysis indicated that GPR176 was significantly enriched in ferroptosis‐related pathways (WP_FERROPTOSIS) and positively correlated with the expression of key ferroptosis‐inhibitory molecules GPX4 and SLC3A2. Figure 1D shows that the protein levels of GPX4 and SLC3A2 were also significantly upregulated in EC tissues with high GPR176 expression. In addition, at the cellular level, Figure 1E shows that GPR176 expression was higher in multiple EC cell lines than in normal esophageal epithelial Het‐1A cells. Taken together, GPR176 is aberrantly overexpressed in EC and closely associated with ferroptosis‐inhibitory signaling, suggesting its potential involvement in ferroptosis regulation in EC.

### 3.2. GPR176 Inhibits Ferroptosis in EC Cells

**Figure 2:**
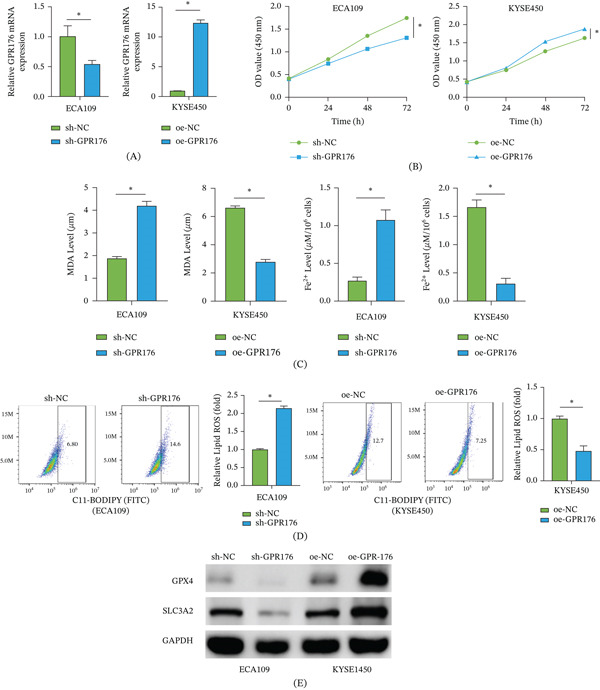
GPR176 inhibits ferroptosis in EC cells Cell groups: sh‐NC, sh‐GPR176, oe‐NC, oe‐GPR176; (A) qRT‐PCR detection of GPR176 mRNA expression in each treatment group of EC cells; (B) CCK‐8 assay for cell viability; (C) Kit‐based detection of MDA and Fe^2+^ levels; (D) Flow cytometry detection of lipid ROS levels in EC cells; (E) WB detection of ferroptosis‐related protein expression (GPX4 and SLC3A2); Data are presented as mean ± SD from three independent experiments. ns, not significant; ∗*p* < 0.05.

Next, we investigated the effect of GPR176 expression changes on the ferroptosis process. Figure 2A shows that GPR176 was successfully knocked down in ECA109 cells and overexpressed in KYSE450 cells, as confirmed by qRT‐PCR. Figure 2B shows that GPR176 knockdown significantly reduced the viability of ECA109 cells, whereas GPR176 overexpression promoted the proliferation of KYSE450 cells. As shown in Figure 2C, among the ferroptosis‐related indicators, GPR176 knockdown led to increased intracellular MDA content and Fe^2+^ levels, while GPR176 overexpression reduced their accumulation. As shown in Figure 2D, flow cytometry demonstrated that GPR176 knockdown promoted the accumulation of lipid ROS in ECA109 cells, whereas its overexpression inhibited lipid ROS generation in KYSE450 cells. Figure 2E shows that GPR176 knockdown decreased the expression of ferroptosis‐inhibitory proteins GPX4 and SLC3A2, while GPR176 overexpression upregulated both proteins. The changes in MDA, Fe2+, lipid ROS, GPX4, and SLC3A2 showed that GPR176 inhibited ferroptosis in EC cells. In conclusion, GPR176 functions as a suppressor of ferroptosis in EC. GPR176 downregulation accelerates ferroptosis, while upregulation inhibits it.

### 3.3. GPR176 Inhibits Mitophagy and Affects Ferroptosis in EC Cells

**Figure 3:**
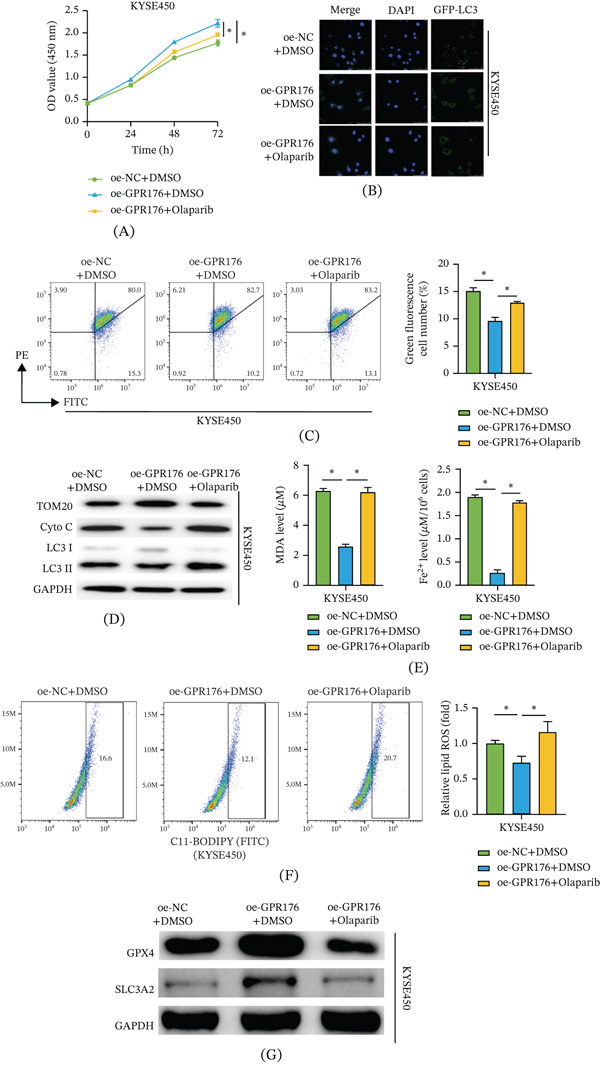
GPR176 inhibits mitophagy and affects ferroptosis in EC cells Cell groups: oe‐NC + DMSO, oe‐GPR176 + DMSO, oe‐GPR176 + olaparib; (A) CCK‐8 assay for cell viability; (B) Immunofluorescence detection of mitophagy in EC cells transfected with GFP‐LC3 plasmid; (C) JC‐1 staining for quantification of MMP changes in EC cells; (D) WB detection of mitochondrial membrane proteins (TOM20 and Cyto C), LC3‐II, and LC3‐I levels; (E) Kit‐based detection of MDA and Fe^2+^ levels in EC cells of each treatment group; (F) Flow cytometry detection of lipid ROS in EC cells of each treatment group; (G) WB detection of ferroptosis‐related protein expression (GPX4 and SLC3A2); Data are presented as mean ± SD from three independent experiments. ns, not significant; ∗*p* < 0.05.

We hypothesized that GPR176 may participate in the suppression of ferroptosis in EC cells by regulating other signaling pathways. Literature reports indicate that GPR176 can promote tumor progression in colorectal cancer by inhibiting mitophagy [19]. To investigate the role of this pathway in ferroptosis of EC cells, the mitophagy‐inducer olaparib was introduced. KYSE450 cells were divided into three groups: oe‐NC + DMSO, oe‐GPR176 + DMSO, and oe‐GPR176 + olaparib. Figure 3A shows that GPR176 overexpression significantly enhanced cell viability, whereas the addition of olaparib markedly reversed this effect. To assess autophagosome formation, KYSE450 cells were transfected with GFP‐LC3. Figure 3B shows that GPR176 overexpression reduced GFP‐LC3 puncta formation, whereas olaparib treatment increased the number of LC3 puncta. These results are consistent with suppressed mitophagy in GPR176‐overexpressing cells. To confirm whether GPR176 overexpression suppresses mitophagy in EC cells, JC‐1 staining was used to assess MMP. Figure 3C shows that GPR176 overexpression increased MMP, whereas olaparib significantly decreased it. Increased MMP suggests reduced mitochondrial depolarization and is consistent with decreased mitophagy‐related mitochondrial turnover. Figure 3D shows that GPR176 overexpression upregulated the mitochondrial membrane protein translocase of the outer mitochondrial membrane 20 (TOM20), while downregulating cytosolic cytochrome c (Cyto C) and the LC3‐II/I ratio; these effects were reversed by olaparib treatment. Increased TOM20 indicates that more mitochondria are preserved. Decreased LC3‐II/I suggests reduced autophagosome formation. Reduced cytosolic Cyto C indicates less mitochondrial damage and release. These changes together show that mitophagy is suppressed by GPR176. Regarding ferroptosis‐related indicators in Figure 3E,F, GPR176 overexpression reduced MDA content, Fe^2+^ levels, and lipid ROS accumulation, whereas olaparib treatment restored these indicators to control levels. The reductions in MDA, Fe2+, and lipid ROS suggest decreased lipid peroxidation and ferroptotic stress. In addition, Figure 3G shows that GPR176 overexpression increased the expression of ferroptosis‐inhibitory proteins GPX4 and SLC3A2, which was significantly attenuated by olaparib treatment. The upregulation of GPX4 and SLC3A2 suggests increased resistance to ferroptosis. Together, GPR176 affects ferroptosis in EC cells by inhibiting mitophagy, which can be reversed by olaparib.

### 3.4. E2F4 Directly Activates GPR176 Transcription in EC

**Figure 4:**
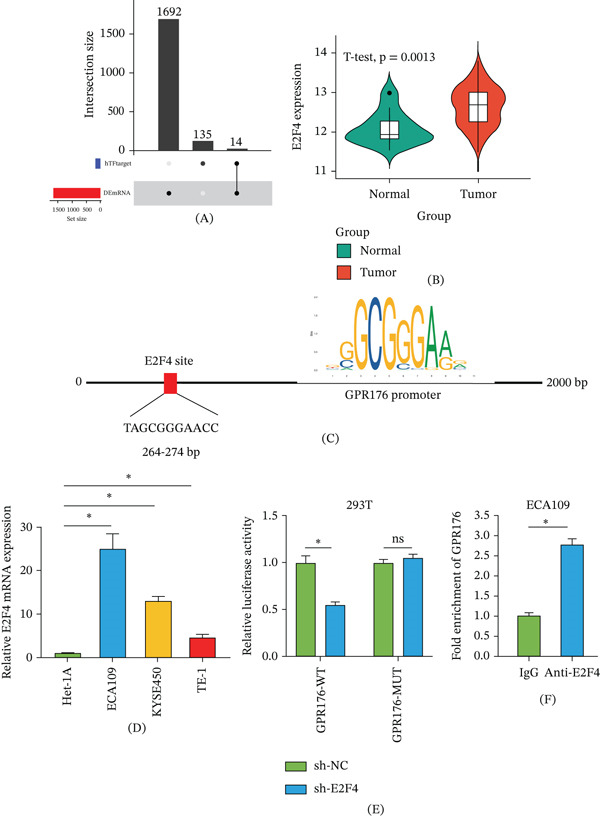
E2F4 directly activates GPR176 transcription in EC. (A) Potential upstream transcription factor E2F4 of GPR176 identified via hTFtarget; (B) Bioinformatic analysis of E2F4 expression in EC tissues; (C) JASPAR prediction of the binding motif between GPR176 and E2F4; (D) qRT‐PCR detection of E2F4 mRNA expression in EC cells of each treatment group; (E) Luciferase reporter assay to detect the interaction between GPR176 and E2F4; (F) ChIP assay detecting binding between GPR176 and E2F4; Data are presented as mean ± SD from three independent experiments. ns, not significant; ∗*p* < 0.05.

To elucidate the upstream regulatory mechanism by which GPR176 inhibits ferroptosis via mitophagy, we predicted potential upstream TFs of GPR176 and identified 14 candidates. As shown in Figure 4A,B, we observed that E2F4 was significantly upregulated in EC tissues. Figure 4C shows that binding site prediction with the JASPAR database identified a specific binding motif between E2F4 and the GPR176 promoter region at 264–274 bp. As shown in Figure 4D, qRT‐PCR analysis confirmed that E2F4 expression was elevated in multiple EC cell lines. Figure 4E shows that E2F4 knockdown significantly reduced the luciferase activity of the wild‐type GPR176 promoter. In addition, Figure 4F shows that E2F4 can directly bind to the promoter region of GPR176 in ChIP analysis. In conclusion, E2F4 directly activates GPR176 transcription in EC cells.

### 3.5. E2F4/GPR176‐Mediated Mitophagy Suppresses Ferroptosis in EC Cells

**Figure 5:**
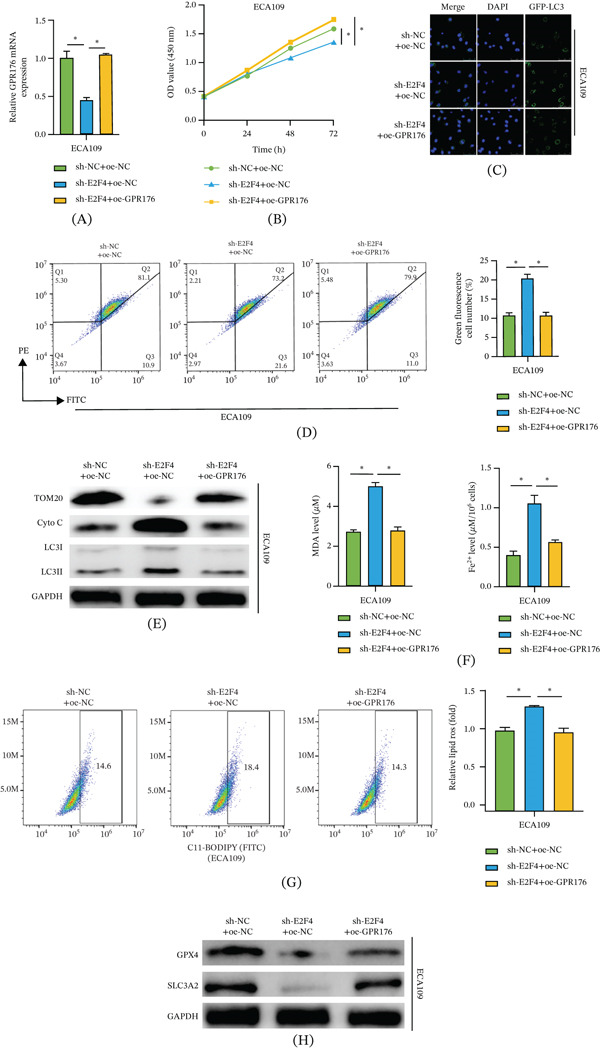
E2F4/GPR176‐mediated mitophagy inhibits ferroptosis in EC cells Cell groups: sh‐NC + oe‐NC, sh‐E2F4 + oe‐NC, sh‐E2F4 + oe‐GPR176; (A) qRT‐PCR detection of GPR176 mRNA expression in EC cells; (B) CCK‐8 assay for cell viability; (C) Immunofluorescence detection of mitophagy in EC cells transfected with GFP‐LC3 plasmid; (D) JC‐1 staining for quantification of mitochondrial membrane potential changes in EC cells; (E) WB detection of mitochondrial membrane proteins (TOM20 and Cyto C), LC3‐II, and LC3‐I; (F) Kit‐based detection of MDA and Fe^2+^ levels in EC cells of each treatment group; (G) Flow cytometry detection of lipid ROS in EC cells of each treatment group; (H) WB detection of ferroptosis‐related protein expression (GPX4 and SLC3A2); Data are presented as mean ± SD from three independent experiments. ns, not significant; ∗*p* < 0.05.

To clarify the E2F4/GPR176 axis in regulating mitophagy and ferroptosis in EC cells, ECA109 cells were divided into three groups: sh‐NC + oe‐NC, sh‐E2F4 + oe‐NC, and sh‐E2F4 + oe‐GPR176. Figure 5A shows that E2F4 knockdown significantly reduced GPR176 mRNA expression, whereas GPR176 overexpression restored its expression in the context of E2F4 knockdown. Figure 5B shows that E2F4 knockdown suppressed cell viability, which was reversed by GPR176 overexpression. Regarding autophagy and mitochondrial function, Figure 5C shows that E2F4 knockdown promoted GFP‐LC3 puncta formation, indicating autophagy activation, whereas GPR176 overexpression inhibited this effect. Figure 5D shows that E2F4 knockdown reduced MMP, whereas GPR176 overexpression restored membrane potential levels. Figure 5E shows that E2F4 knockdown decreased TOM20 expression and increased the LC3‐II/I ratio and cytosolic Cyto C levels, while GPR176 overexpression reversed these protein expression changes. Reduced TOM20 indicates loss of mitochondrial mass, increased LC3‐II/I reflects autophagy activation, and elevated cytosolic Cyto C suggests mitochondrial damage, collectively indicating enhanced mitophagy. As shown in Figure 5F,G, ferroptosis‐related phenotypic assays demonstrated that E2F4 knockdown increased MDA content, Fe^2+^ levels, and lipid ROS accumulation, whereas GPR176 overexpression suppressed these changes. These changes indicate enhanced lipid peroxidation and oxidative stress, which are hallmarks of ferroptosis. Figure 5H shows that E2F4 knockdown decreased the expression of ferroptosis‐inhibitory proteins GPX4 and SLC3A2, whereas GPR176 overexpression restored their protein levels, suggesting reduced resistance to ferroptosis. The changes in MDA, Fe2+, lipid ROS, GPX4, and SLC3A2 indicate that E2F4 knockdown promotes ferroptosis, while GPR176 overexpression reverses this effect. In summary, E2F4 transcriptionally activates GPR176, inhibiting mitophagy and reducing ferroptosis sensitivity in EC cells. GPR176 overexpression can effectively rescue the mitophagy activation and enhanced ferroptosis phenotype caused by E2F4 knockdown.

## 4. Discussion

Despite substantial advancements in EC management, existing therapeutic strategies continue to demonstrate limited efficacy for individuals with recurrent or resistant conditions. Enhancing patient outcomes therefore represents a pressing and unresolved clinical priority [4]. Therefore, it is of great significance to elucidate the driving mechanisms underlying EC recurrence and progression and to explore effective therapeutic targets. In the present study, we combined bioinformatics, clinical tissue analysis, and in vitro experiments to demonstrate the pivotal involvement of the TF E2F4 and its target gene GPR176 in EC progression. This study demonstrates that E2F4 transcriptionally upregulates GPR176 in EC cells, which inhibits mitophagy and thereby promotes ferroptosis resistance. This newly identified ferroptosis resistance pathway in EC, linking transcriptional control to organelle function, presents a potential therapeutic target for preventing EC recurrence.

Belonging to the GPCR family, GPR176 functions as an oncogene in several malignancies, such as gastric, colorectal, and breast cancer [15, 20, 21]. Notably, it is also highly expressed in EC. Yun et al. reported that GPR176 drives tumor progression and induces chemoresistance in EC by activating the ACC1/ACLY lipid synthesis pathway, which is associated with poor patient prognosis [22]. This aligns with the present study, which confirms GPR176’s high expression in EC and its involvement in tumor ferroptosis. Notably, in colorectal cancer, GPR176 suppresses mitophagy through the cAMP/PKA/BNIP3L signaling cascade, facilitating cancer advancement [19]. This mechanism suggests the contribution of GPR176 to tumor cell homeostasis by regulating mitophagy; however, its regulatory role in ferroptosis and the mitophagy pathway in EC remains unexplored.

Ferroptosis, as a novel regulated form of cell death, has received considerable attention in tumor suppression research [23]. Given that GPCR family members suppress ferroptosis through downstream signaling activation [18, 24, 25], we hypothesized that GPR176 might possess a comparable role. In line with this, our findings demonstrate that GPR176 is upregulated in EC and indeed acts to block ferroptosis, supporting tumor survival. This was supported by the consistent changes in MDA, Fe2+, lipid ROS, GPX4, and SLC3A2 in our cell experiments. The correlation between mitophagy and ferroptosis is known to vary across contexts [13]. For instance, simvastatin has been observed to protect pancreatic cancer cells from ferroptosis through mitophagy induction [26]; in liver cancer, plant‐derived nanovesicles induce mitophagy but synergistically exacerbate ferroptosis by releasing iron ions and promoting lipid peroxidation [27]. These seemingly contradictory findings suggest that mitophagy may exert bidirectional regulatory effects in ferroptosis. Our research reinforces the ferroptosis‐promoting function of mitophagy. We observed that GPR176 appears to increase resistance to ferroptosis in EC cells through the suppression of mitophagy, which decreases iron release and lipid peroxidation linked to mitochondrial breakdown. Therefore, targeting the GPR176‐mediated mitophagy suppression pathway may provide a novel therapeutic direction for EC. In our study, pharmacological activation of mitophagy with olaparib reversed the inhibitory effects of GPR176 on MDA, Fe2+, lipid ROS, GPX4, and SLC3A2, supporting the view that mitophagy activation facilitates ferroptosis in EC cells.

To investigate the upstream mechanism underlying the aberrant high expression of GPR176 in EC, we identified the TF E2F4 through bioinformatics analysis. E2F4 belongs to the E2F family of TFs and functions by binding to particular promoter sequences to modulate the transcription of its target genes [28]. E2F4 is highly expressed in multiple malignancies, including prostate cancer, gastric cancer, and liver cancer [29–31], and functions as a key oncogenic TF in EC [32]. Notably, in colorectal cancer, MTCH2 deficiency promotes E2F4 degradation, relieving E2F4‐mediated transcriptional repression of the ferroptosis‐related gene TFRC, which activates the ferroptosis pathway and enhances the efficacy of sorafenib [33]. This suggests that E2F4 may function as a suppressor of ferroptosis. However, the involvement of E2F4 in modulating ferroptosis and mitophagy in EC is not well understood. Our experiments demonstrated that silencing E2F4 in EC cells significantly increases both mitophagy and ferroptosis, which can be counteracted by overexpressing GPR176. These findings suggest that E2F4 supports EC progression by suppressing mitophagy and ferroptosis, revealing a new mechanism that could inform therapeutic approaches aimed at inducing ferroptosis in EC. This oncogenic role of E2F4 in EC may be related to its ability to activate downstream target genes that promote tumor cell survival. In this study, E2F4 upregulates GPR176, which inhibits mitophagy and suppresses ferroptosis. This leads to enhanced cell survival and contributes to tumor progression.

In summary, the E2F4/GPR176 axis increases ferroptosis resistance in EC cells by suppressing mitophagy. The study, however, has certain limitations. Firstly, the findings are mainly derived from cellular experiments and await validation in animal models. Secondly, the downstream molecular network regulated by GPR176, especially the precise connections between mitophagy and ferroptosis, needs clarification. Future work will involve in vivo studies to confirm the functional relevance of this axis and to map out the complete E2F4/GPR176/mitophagy molecular network governing ferroptosis. This deeper understanding aims to offer a more translatable foundation and targeted strategy for EC therapy.

## Author Contributions

Conceived and designed the experiments: Wenfa Lin, Zaiyuan Ye.

Analyzed the data: Tao Ding, Xiao Qi, Peiqiang Chen.

Wrote and revised the manuscript: Weilang Xu, Fang Su, Luxuan Xie.

Draw figures: Tao Ding, Xiao Qi, Peiqiang Chen.

Finish experiments: Weilang Xu, Fang Su, Luxuan Xie.

Weilang Xu and Fang Su contributed equally.

## Funding

No funding was received for this manuscript.

## Ethics Statement

This study was approved by the ethics committee of the Shulan (Hangzhou) Hospital in accordance with the Declaration of Helsinki. All patients voluntarily participated and provided written informed consent.

## Conflicts of Interest

The authors declare no conflicts of interest.

## Supporting information


**Supporting Information** Additional supporting information can be found online in the Supporting Information section. Table S1: Sequences of qRT‐PCR primers used for detecting GPR176, E2F4, and *β*‐actin expression levels in human EC cells. Table S2: List of primary and secondary antibodies used for Western blot analysis, including catalog numbers and manufacturers. Table S3: List of primary and secondary antibodies used for immunohistochemistry, including catalog numbers and manufacturers. Table S4: Sequences of ChIP‐qPCR primers used to analyze E2F4 binding to the GPR176 promoter.

## Data Availability

All data generated or analyzed during this study are included in this published article and are available from the corresponding author on reasonable request.
