# Supplementary material for: E2F4 Mediates Mitophagy to Inhibit Ferroptosis in Esophageal Cancer Cells by Activating GPR176
Source: Hum Mutat. 2026 May 30;2026:9418012. doi: 10.1155/humu/9418012 (PMC13239033; doi:10.1155/humu/9418012)
Supplement: Supplementary file 1 — Supporting Information Additional supporting information can be found online in the Supporting Information section. Table S1: Sequences of qRT‐PCR primers used for detecting GPR176, E2F4, and β‐actin expression levels in human EC cells. Table S2: List of primary and secondary antibodies used for Western blot analysis, including catalog numbers and manufacturers. Table S3: List of primary and secondary antibodies used for immunohistochemistry, including catalog numbers and manufacturers. Table S4: Sequences of ChIP‐qPCR primers used to analyze E2F4 binding to the GPR176 promoter. [file HUMU-2026-9418012-s001.docx]

Supplementary Table 1: Sequences of qRT-PCR primers

| Gene | Primer sequence (5’→3’) |
| --- | --- |
| GPR176  (*Homo sapiens*) | F: TCTTCATAGGCTCGCTGCTC |
|  | R: AATGAACTTAATCCCAGGCAGT |
| E2F4  (*Homo sapiens*) | F: CCCCTACAGAGCTTGAGAGC |
|  | R: CAAACACTTTGGCTCCACGG |
| β-actin  (*Homo sapiens*) | F: CCAAAGTTCACAATGTGGCCG |
|  | R: TGGACTTGGGAGAGGACTGG |

Supplementary Table 2: Antibodies for Western blot

| Antibody | Catalog Number | Manufacturer |
| --- | --- | --- |
| Rabbit anti-GPX4 antibody | A27995 | ABclonal, USA |
| Rabbit anti-SLC3A2 antibody | A28528 | ABclonal, USA |
| Rabbit anti-LC3B antibody | A19665 | ABclonal, USA |
| Rabbit anti-TOM20 antibody | A19403 | ABclonal, USA |
| Rabbit anti-Cyto C antibody | A4912 | ABclonal, USA |
| Rabbit anti-GAPDH antibody | A19056 | ABclonal, USA |
| Goat anti-Rabbit IgG H&L (HRP) antibody | ab205718 | Abcam, UK |

Supplementary Table 3: Antibodies for immunohistochemistry

| Antibody | Catalog Number | Manufacturer |
| --- | --- | --- |
| Rabbit anti-GPR176 antibody | bs-15387R | BIOSS, China |
| Rabbit anti-GPX4 antibody | A27995 | ABclonal, China |
| Rabbit anti-SLC3A2 antibody | 15193-1-AP | Proteintech, China |
| Goat anti-Rabbit IgG H&L (HRP) antibody | ab205718 | Abcam, UK |

Supplementary Table 4: Sequences of ChIP-qPCR primers

| Gene | Primer sequence (5’→3’) |
| --- | --- |
| GPR176 | F: CTGGGCTAAGGGGAGACAAG |
|  | R: AATGAAACATCCCCCGGCTT |
